# Liquid-Gated Field-Effect Transistor-Based Biosensor for Uric Acid Detection

**DOI:** 10.3390/bios16030142

**Published:** 2026-03-01

**Authors:** Rafiq Ahmad, Abdullah, Altaf Khan, Fohad Mabood Husain, Byeong-Il Lee

**Affiliations:** 1 New-Senior’ Oriented Smart Health Care Education Center, Pukyong National University, Busan 48513, Republic of Korea; 2 Future Energy Convergence Core Center, Jeonbuk National University, Jeonju 54896, Republic of Korea; abdullahazmi@jbnu.ac.kr; 3 Department of Pharmacology, College of Pharmacy, King Saud University, Riyadh 11451, Saudi Arabia; altkhan@ksu.edu.sa; 4 Department of Food Science and Nutrition, College of Food and Agriculture Sciences, King Saud University, Riyadh 11451, Saudi Arabia; fhusain@ksu.edu.sa; 5 Industry 4.0 Convergence Bionics Engineering, Pukyong National University, Busan 48513, Republic of Korea; 6 Digital Healthcare Research Center, Institute of Information Technology and Convergence, Pukyong National University, Busan 48513, Republic of Korea; 7 Division of Smart Healthcare, College of Information Technology and Convergence, Pukyong National University, Busan 48513, Republic of Korea

**Keywords:** field-effect-transistor, ZnO nanorods, biosensor, uric acid, serum sample

## Abstract

Monitoring uric acid (UA) concentration is crucial for human health, enabling early detection and prevention of metabolic disorders as well as assessing renal function and overall metabolic balance. Herein, we developed a field-effect transistor (FET)-based UA biosensor using hydrothermally synthesized vertical zinc oxide (ZnO) nanorods (NRs) and uricase. The fabricated FET biosensor was tested in phosphate-buffered saline (PBS) at increasing UA concentrations to evaluate its biosensing performance. The FET biosensor yields a sensitivity of 12.45 μA·mM^−1^·cm^−2^, covering a dynamic range of 0.05–2.75 mM. The calculated detection limit was ~0.0043 mM. The improved sensing performance results in a substantial enhancement of both detection sensitivity and limit of detection compared to the traditional lateral electrode setup. Additionally, selectivity, storage stability, fabrication reproducibility, and applicability for serum UA detection were evaluated. Overall, the vertical electrode configuration of the UA biosensor has the potential to be further extended for the sensitive detection of additional biomarkers.

## 1. Introduction

Small biomolecules such as uric acid (UA) are vital to numerous biochemical and physiological processes in the human body [1]. UA compound is naturally present in the extracellular fluids of the central nervous system (CNS) and in blood serum, where it contributes significantly to maintaining normal metabolic and neurological functions [2]. UA is the end product of the purine metabolic pathway in humans [3]. It is primarily eliminated from the body through urine, feces, and perspiration. Maintaining an optimal UA concentration is crucial because deviations, particularly elevated levels, can lead to metabolic and renal complications, including hyperuricemia, gout, gouty arthritis, anaemia, and nephropathy [4,5]. Given the physiological relevance and clinical implications of abnormal UA concentrations, accurate and rapid UA detection is of great importance. Such analytical capability is valuable for medical diagnostics and therapeutic monitoring, particularly in studies of metabolic disorders.

The quantitative determination of UA is traditionally performed by analysing blood or urine samples using several well-established laboratory techniques [6,7,8]. These include liquid chromatography, colorimetric assays, mass spectrometry, capillary electrophoresis, fluorescence spectroscopy, and ultraviolet (UV) spectrophotometry. While these analytical methods are recognized for their high accuracy and reliability, they often suffer from significant drawbacks [9]. Common limitations include lengthy, complex sample preparation, high operational costs, the need for skilled personnel, and the inability to perform rapid or continuous monitoring [10]. As a result, they are less suitable for real-time or on-site clinical diagnostics. In contrast, electrochemical detection techniques have emerged as a fast, cost-effective, and highly adaptable alternative [11,12,13]. These methods offer simple operation, short analysis time, and compatibility with miniaturized electronic systems, making them ideal for developing portable and point-of-care (POC) diagnostic devices. Moreover, integrating biosensing components, such as enzymes or nanostructured materials, further enhances the sensitivity and selectivity of electrochemical detection systems [14,15]. This combination enables UA measurement even at low concentrations, providing an efficient platform for real-time biochemical monitoring and medical diagnostics.

Recently, various innovative biosensor architectures have been developed for the specific detection of individual biomolecules in biofluids, primarily to improve the diagnosis and management of various diseases [16,17,18]. These biosensors have demonstrated remarkable advances in precision, sensitivity, and analytical reliability, enabling health monitoring. Among the different biosensing approaches, field-effect transistor (FET) biosensors have attracted significant interest for their high sensitivity, rapid response, and ability to transduce biochemical changes into electrical signals [19,20]. In these systems, the analyte’s binding to the modified FET channel surface alters the local charge environment, which is detected as a change in the device’s electrical output. Traditionally, many FET biosensors based on Si nanowires and graphene have been reported, offering enhanced surface-to-volume ratios and superior signal transduction efficiency compared with bulk materials [20,21,22,23,24]. These studies have demonstrated high sensitivity, but face limitations such as complex fabrication, surface instability, and poor reproducibility. Si-FETs require costly lithography, while graphene FETs often suffer from inconsistent functionalization and signal variation. Other emerging 2D materials and CMOS-compatible FETs also show promise but still face challenges in large-scale integration and long-term stability. These limitations highlight the need for FET architectures that offer high performance together with simple, reproducible, and scalable fabrication. During FET-based biosensor fabrication, channel modification and gate modification are two key functionalization approaches [25,26,27]. In channel modification, sensing elements are directly attached to the semiconductor channel, enabling strong interaction with charge transport and providing high sensitivity and rapid response. However, this approach may alter intrinsic channel properties, thereby increasing device variability and reducing long-term stability. In contrast, gate modification involves functionalizing the gate electrode while leaving the channel unchanged, thereby maintaining stability, reproducibility, and durability. Nevertheless, signal transmission through the electrolyte and capacitance effects may sometimes result in lower sensitivity and slower response compared to channel modification. Overall, channel modification is generally favored when maximum sensitivity and rapid signal generation are required, whereas gate modification is advantageous for achieving higher reproducibility, operational robustness, and extended device lifespan. The selection between these two strategies depends on the targeted sensing application and required performance characteristics.

To improve the sensing performance and stability of the bioelectrode, researchers have considered zinc oxide (ZnO) as a leading material for biosensor fabrication [28,29,30]. One of the significant advantages of ZnO is its ability to be directly synthesized as well-aligned nanostructures—such as nanorods (NRs), nanowires, or nanotubes—on a variety of substrates or electrodes through cost-effective and straightforward hydrothermal techniques [29,31,32,33,34,35,36]. This direct growth not only simplifies device fabrication but also enhances mechanical adhesion and electrical contact, leading to biosensors with high sensitivity, long-term stability, and superior reproducibility. Also, a unique feature of ZnO is its elevated isoelectric point (IEP) of approximately 9.5, which makes it particularly suitable for the immobilization of biomolecules or enzymes with lower IEP values, such as uricase (*Candida* sp.; IEP ≈ 5.6), via electrostatic adsorption under physiological pH conditions. The vertically oriented ZnO nanostructures offer unique structural and physicochemical properties, making them highly effective for biosensing applications [20]. Their vertically aligned architecture provides efficient charge transport and improved analyte accessibility to the electrode surface. Moreover, the high surface area and aspect ratio of ZnO NRs increase the number of active binding sites, thereby enhancing sensing sensitivity. Their alignment also promotes rapid electron transfer, thereby enhancing signal transduction and electrochemical response, thereby improving overall sensor performance and reliability. Mainly, an extensive surface-to-volume ratio is crucial for improving the sensitivity, response time, and overall performance of biosensors, making ZnO an exceptional candidate for next-generation bioelectronic and diagnostic devices.

Here, we report a liquid-gated FET-based UA biosensor employing vertically aligned ZnO NRs, synthesized via a highly reproducible hydrothermal growth process. Before electrochemical measurements, uricase was immobilized on the vertically grown ZnO NR channel via physical adsorption, enabling specific and efficient detection of UA. The fabricated liquid-gated FET-based UA biosensor demonstrated excellent sensitivity, a broad linear detection range extending up to 2.75 mM, and a low detection limit. The device’s superior sensing capability is primarily attributed to the well-ordered vertical alignment of ZnO NRs, which provides a high surface-to-volume ratio and abundant active sites for enzyme attachment, thereby facilitating electron transfer and catalytic activity. Furthermore, the selectivity, fabrication reproducibility, stability, and applicability of the biosensor for serum UA detection were systematically evaluated, confirming its potential for real-sample UA analysis and biomedical diagnostic applications.

## 2. Materials and Methods

### 2.1. Chemicals

UA (≥99%), Zinc nitrate hexahydrate (Zn(NO_3_)_2_·6H_2_O, 99%), cholesterol (Sigma grade), hexamethylenetetramine (HMTA, 99%), lactose, urea (analytical standard), glucose (D-(+)-glucose (≥99.5%)). Uricase from *Candida* sp., ascorbic acid (AA), Nafion (Nf; 5 wt.%), dopamine (DA; ≥98%), phosphate-buffered saline (PBS) solution (pH 7.0), and serum (human blood; H4522) were from Sigma-Aldrich (Burlington, MA, USA). These were used as-received. Polydimethylsiloxane (PDMS) was prepared using Dow Sylgard 184 silicone elastomer obtained from Dow Europe GmbH (Horgen, Switzerland). Ultrapure deionized (DI) water (18.2 MΩ·cm) was used for all solution preparation and in material synthesis.

### 2.2. Fabrication Steps of FET-Based UA Biosensor

**Scheme 1:**
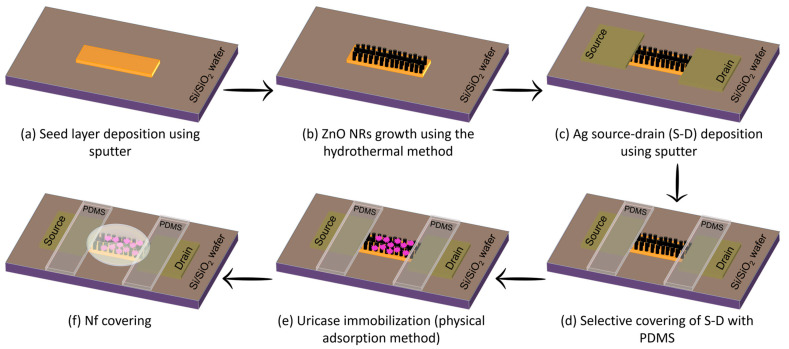
Schematic illustration of FET-based UA biosensor fabrication.

A schematic of the FET-based UA biosensor is illustrated in Scheme 1. To fabricate the FET-based biosensor, a silicon (Si) substrate with a thermally grown silicon dioxide (SiO_2_) dielectric layer was used as the base platform. During FET biosensor fabrication, a thin (~60 nm) ZnO layer was first radio frequency (RF)-sputtered directly onto a Si/SiO_2_ substrate (a). Next, using a hydrothermal process, vertical ZnO NRs were formed on a seeded Si/SiO_2_ substrate (b). In brief, equimolar solutions of Zn(NO_3_)_2_·6H_2_O (0.05 M) and HMTA were mixed in 50 mL DI water and placed in a Pyrex glass container. The seeded substrates were then suspended upside down in the solution and incubated at 80 °C for 3 h in a laboratory oven. After growth, electrodes were purified by washing with DI water before subsequent characterization. Then, silver (Ag; ~100 nm) source-drain (S-D) electrodes were sputtered (c). The Ag electrode-ZnO NRs joining area was covered with PDMS, leaving a 6 mm^2^ active sensing region exposed (d). This passivation minimized leakage currents and eliminated contributions from the metal–nanorod contact areas, ensuring that conductance variations originated solely from the ZnO NRs. In the next step, ZnO NRs-grown electrodes were PBS-washed to enhance surface hydrophilicity, then uricase was immobilised (e). The uricase solution (5 μL; 10 U/mL) was physically adsorbed onto ZnO NRs and incubated overnight at 4 °C. After 12 h, the biosensor electrodes were rinsed with PBS to remove unbound enzyme. Finally, a 0.5% Nf was applied to prevent enzyme leakage and reduce interference from extraneous species (f).

### 2.3. Structural Characterization of ZnO NRs and FET Biosensor Measurements

The structure and crystallinity of ZnO NRs were analyzed via FESEM (field emission scanning electron microscope; Hitachi High-Tech Corporation, Tokyo, Japan). Energy-dispersive spectroscopy (EDS) coupled with FESEM was used to determine elemental composition. Structural properties were studied using X-ray diffraction (XRD; Rigaku Smart Lab, Rigaku Corporation, Tokyo, Japan) with a 2θ range of 30–65° and a scan rate of 8°/min. In addition, Raman spectroscopy (Renishaw Centrus, Renishaw plc, Gloucestershire, UK) was used to examine ZnO NRs’ phase and chemical structure. The fabricated biosensor was coupled with an HP 4155A analyzer (Hewlett-Packard, Palo Alto, CA, USA) for real-time electrical analysis. Device characterization was carried out using two-terminal FET measurements under a solution-gated configuration. The electrical conductance of the biosensor was evaluated by applying a fixed drain-to-source voltage (V*_ds_*) and monitoring the resulting drain current (I*_ds_*) at various UA concentrations. To ensure reproducibility, each sensing experiment was performed with 3–4 independently fabricated biosensors, all of which exhibited consistent electrical responses.

## 3. Results and Discussion

### 3.1. Characterization of Vertical ZnO NRs

**Figure 1:**
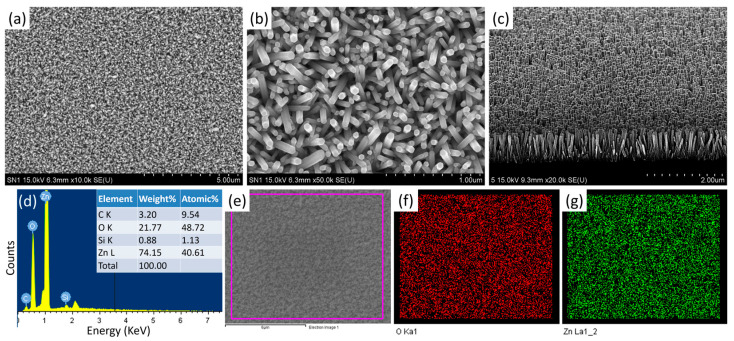
FESEM showing top (**a**,**b**) and cross-view (**c**), EDS (**d**), and elemental mapping (**e**–**g**) of vertically grown ZnO NRs on SiO_2_-Si substrate.

Surface and cross-view morphologies of the vertical ZnO NRs grown on the seeded Si/SiO_2_ substrates were examined using FESEM, as presented in Figure 1. The surface images (Figure 1a,b) at low and high magnifications indicate that the ZnO NRs are uniformly covering a wide area at high density. The cross-sectional image (Figure 1c) further confirms that the nanorods are densely packed and vertically oriented on the substrate surface. The nanorods have an average length of approximately 960 ± 10 nm and a diameter of 75 ± 5 nm, yielding a high aspect ratio. The high aspect ratio significantly increases the effective surface area available for enzyme immobilization. The larger surface-to-volume ratio facilitates higher enzyme loading, enhances electron-transfer efficiency, and ultimately improves the sensitivity and overall biosensing performance of the FET device. The elemental composition of the vertical ZnO NRs was examined using EDS (Figure 1d–g). Elemental composition analysis of the ZnO NRs confirmed the presence of zinc (Zn), oxygen (O), carbon (C), and silicon (Si) as the detected elements, with their respective atomic percentages of 40.61%, 48.72%, 9.54%, and 1.13% (Figure 1d). The carbon and silicon signals observed in the EDS spectrum primarily originate from the carbon adhesive tape and silicon substrate, respectively. Elemental mapping further illustrates the homogeneous distribution of Zn and O (Figure 1e–g). These results are consistent with the expected elemental composition, confirming successful synthesis of pure vertical ZnO NRs.

**Figure 2:**
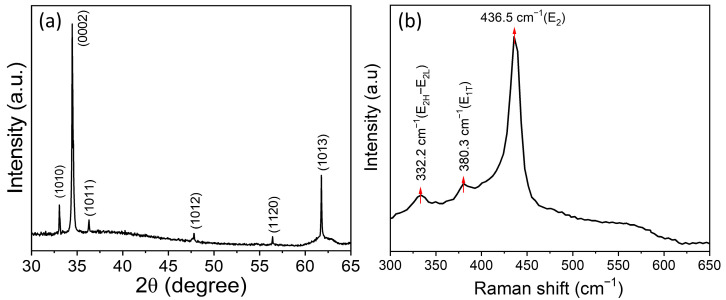
Typical XRD pattern (**a**) and Raman spectrum (**b**) of as-synthesized ZnO NRs.

XRD analysis characterized the crystal phase and growth orientation of ZnO NRs (Figure 2a). All diffraction peaks correspond to the hexagonal wurtzite phase of ZnO (JCPDS: 75-1526) [37]. The dominant peak observed at 34.2°, indexed to the (0002) plane of ZnO, verifies that the as-grown ZnO NRs exhibit a preferential orientation along the [0001] direction. Raman spectroscopy was used to analyze ZnO NRs’ structural properties further, as shown in Figure 2b. A sharp peak at 436.5 cm^−1^ corresponds to the E_2_ (high) Raman mode of wurtzite ZnO [38]. Additionally, two weaker peaks located at 332.2 cm^−1^ and 380.3 cm^−1^ are attributed to the E_2_H–E_2_L (multi-phonon process) and A_1_(T) modes, respectively [39]. No peak corresponding to the E_1_(L) mode is observed in the spectrum. The E_1_(L) denotes structural defects such as oxygen vacancies or zinc interstitials [34]. Therefore, an intense and well-defined E_2_ mode, along with the absence of the E_1_(L) peak, confirms that the synthesized ZnO NRs possess excellent crystalline quality with a hexagonal wurtzite structure.

### 3.2. Detection of UA Using FET Biosensor

**Figure 3:**
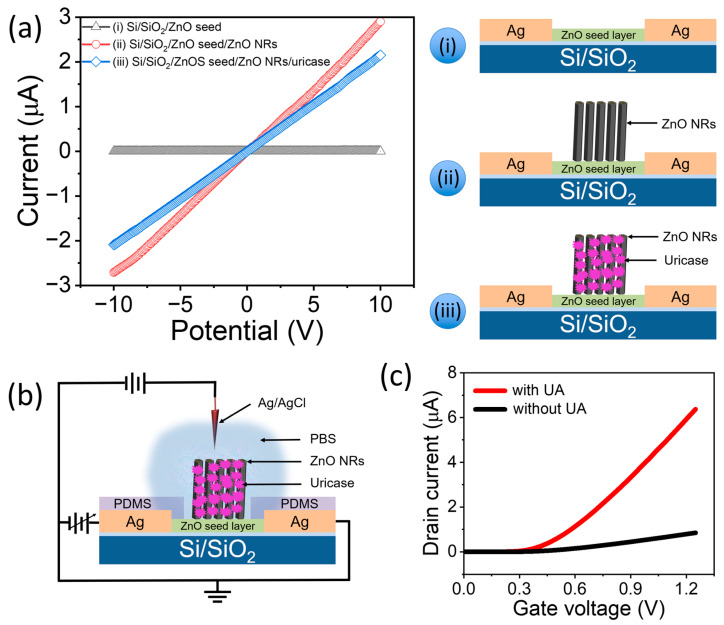
(**a**) Typical I-V curves of the Si/SiO_2_ substrate FET with (**i**) ZnO seed layer, (**ii**) ZnO seed layer/ZnO NRs, and (**iii**) ZnO seed layer/ZnO NRs/uricase, along with respective schematics, (**b**) overall schematic configuration of the FET-based UA biosensor, and (**c**) *I*_D_–*V*_G_ response of the FET biosensor in PBS buffer without and with 0.1 mM UA (applied Vds = 0.1 V).

The I–V characteristic of the FET sensor was measured at each fabrication step to demonstrate the effect of modification. The typical I–V curves of the Si/SiO_2_ substrate FET with (i) ZnO seed layer, (ii) ZnO seed layer/ZnO NRs, and (iii) ZnO seed layer/ZnO NRs/uricase are shown in Figure 3a, along with their respective schematics. The ZnO seed-layer-based FET device showed a lower current response than the other two FET devices (i.e., ZnO seed layer/ZnO NRs and ZnO seed layer/ZnO NRs/uricase). After uricase immobilization onto ZnO NRs, the I–V characteristics of the FET device exhibit an apparent decrease in drain current and channel conductance. This reduction arises from the negatively charged enzyme layer, which modulates the ZnO surface potential and depletes surface electrons in the *n*-type channel [40,41]. The shift in I–V response confirms successful enzyme attachment and indicates that the biofunctionalized ZnO NRs are actively influencing the FET’s electrical behavior.

The liquid-gated FET-based UA biosensor is shown in Figure 3b. In this configuration, an Ag/AgCl reference electrode is immersed in the electrolyte, which serves as the liquid-ion-gate. The Ag/AgCl reference electrode establishes an electrostatic gate potential across the electrolyte, in which the applied gate voltage induces the formation of an electric double layer (EDL) at the electrolyte/ZnO interface [42]. This EDL acts as an ultra-thin, high-capacitance dielectric layer. The modulation of ionic charges in the electrolyte is electrostatically coupled to the surface of the ZnO NRs, which in turn alters the electron concentration in the *n*-type ZnO channel [43]. However, there is no direct electrical connection to the semiconductor. This allows slight variations in surface charge (such as UA oxidation catalyzed by uricase) to produce measurable changes in channel conductance.

Figure 3c presents the transfer characteristics (*I*_D_–*V*_G_ curves) of the liquid-gated FET-based UA biosensor exposed to a 0.1 mM UA solution. The data clearly demonstrate that the biosensor with UA in PBS buffer exhibits a markedly higher drain current response than that measured in PBS buffer alone. The enhanced current response results from the enzymatic oxidation of UA catalyzed by uricase immobilized on the ZnO NR surface. During this reaction, UA undergoes oxidation to produce allantoin, carbon dioxide (CO_2_), and hydrogen peroxide (H_2_O_2_) [44,45,46]. Subsequently, enzymatic oxidation generates reaction products (e.g., H_2_O_2_, followed by protons and electrons) at the ZnO NR surface. These processes alter the local surface potential by modifying the charge distribution at the semiconductor–electrolyte interface. The accumulation of released electrons in the ZnO channel increases carrier concentration, thereby shifting the threshold voltage toward lower gate potentials and enhancing drain current, as observed in the transfer characteristics (Figure 3c). Simultaneously, the electrical double layer (EDL) formed at the ZnO–electrolyte interface plays a crucial role in signal amplification. Variations in ionic concentration and interfacial charge density during the enzymatic reaction modulate the EDL capacitance, thereby influencing the gate coupling efficiency. This improved charge modulation enhances transconductance and overall device sensitivity. Furthermore, changes in the effective surface charge arising from biomolecular adsorption and enzymatic by-products directly affect channel conductance via electrostatic doping of *n*-type ZnO NRs [47]. However, excessive surface modification or prolonged biochemical interactions may introduce interfacial trap states or scattering centers that can affect carrier mobility and long-term stability. Overall, the sensing mechanism is governed by interfacial electrostatic modulation, in which changes in surface potential, EDL structure, and effective surface charge collectively regulate the threshold voltage, transconductance, and drain current, thereby determining device performance. Consequently, the observed increase in current can be directly attributed to charge transfer from the enzymatic by-products to the ZnO channel, thereby enhancing the biosensor’s sensitivity to UA detection.

**Figure 4:**
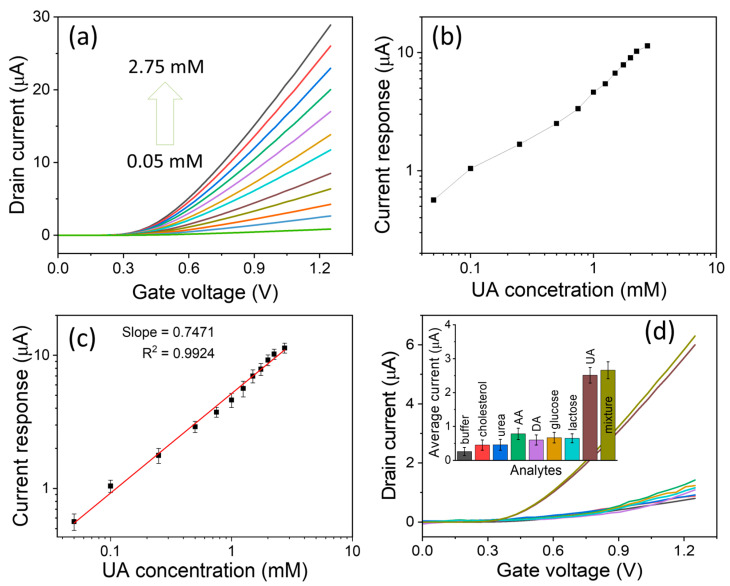
Sensing performance of FET-based UA biosensors. (**a**) *I*_D_–*V*_G_ response of liquid-gated FET UA biosensor with increasing UA concentrations, (**b**) plot of current vs. UA concentration, (**c**) linear range calibration plot, and (**d**) selectivity test. Inset (**d**) shows the average current-response histogram for four similar fabricated FET-based UA biosensors.

Figure 4a illustrates the transfer characteristics (*I*_D_–*V*_G_ curves) of the uricase-functionalized ZnO NR-based liquid-gated FET-based UA biosensor recorded at a range of UA concentrations. As shown in Figure 4a, the biosensor exhibits an increasing drain current with increasing UA concentration from 0.05 to 2.75 mM. A significant enhancement in current is particularly evident within the gate voltage range of +0.3 to +1.25 V. This behavior is primarily associated with the generation of H_2_O_2_ as a byproduct of the enzymatic oxidation of UA catalyzed by uricase in the presence of dissolved oxygen in the PBS solution [48]. As the UA concentration increases, the drain current response rises correspondingly and eventually saturates at higher concentrations. Figure 4b shows the plot of current response versus UA concentration, obtained from the biosensor’s average current values recorded over a gate voltage range of +0.3 to +1.25 V. After repeating the experiment with 3 similar liquid-gated FET-based UA biosensors, their response calibration curve was plotted (Figure 4c). From the calibrated plot, the biosensor demonstrates an excellent linear response over a range of 0.05–2.75 mM (correlation coefficient, R^2^ = 0.9924), indicating high linearity and measurement precision. Notably, this broad linear detection range surpasses that of many previously reported UA biosensors (Table 1) [49,50,51,52,53,54,55,56,57]. The extended linear range is mainly due to dense, vertically aligned ZnO NRs, which provide a large surface area for high enzyme (uricase) loading, thereby enhancing the device’s catalytic activity and signal transduction efficiency. The biosensor sensitivity was calculated to be 12.45 μA·mM^−1^·cm^−2^ from the slope of the calibration curve. The limit of detection (LOD) was estimated at ~0.0043 mM using LOD = 3.3(SD/S), where SD is the response standard deviation, and S is the calibration slope. The comparatively higher LOD observed can be attributed to differences in sensor architecture, material morphology, and fabrication parameters compared to previously reported methods listed in Table 1. In the current design, the sensing performance is primarily influenced by the device channel dimensions and the intrinsic conductivity of the ZnO nanostructure. To further improve the detection sensitivity and reduce the LOD, several optimization strategies can be implemented. For instance, reducing the channel length can significantly enhance charge carrier transport and improve signal amplification. Additionally, surface modification of ZnO nanostructures, such as doping, functionalization with catalytic materials, or hybrid nanocomposite formation, can enhance electrical conductivity and increase the density of active sensing sites. These approaches are expected to improve electron transfer efficiency and ultimately lower the detection limit.

### 3.3. Selectivity, Reproducibility, and Stability Tests

The liquid-gated FET-based UA biosensor’s selectivity was evaluated by examining its response to various potential interfering substances. The test was conducted using individual solutions containing the interferents (0.1 mM each)—cholesterol, urea, AA, DA, glucose, and lactose)—as well as a 0.1 mM UA solution. Additionally, a mixed solution containing 0.1 mM UA combined with all interfering species was tested, as illustrated in Figure 4d. The corresponding current responses are summarized in a histogram (inset, Figure 4d). The results show that all interfering species produced minimal changes in current relative to the strong signal observed for UA alone, indicating low cross-reactivity. Notably, even in the presence of all potential interferents, the biosensor maintained a distinct and selective response to UA, confirming its excellent specificity and resistance to interference.

**Figure 5:**
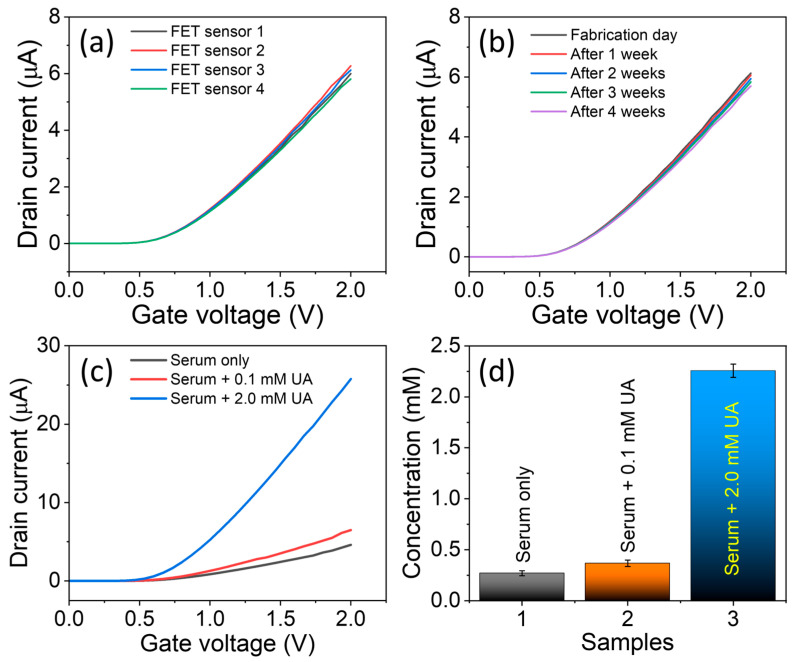
Reproducibility, stability, and real sample analysis. (**a**) *I*_D_–*V*_G_ response of the four similar fabricated liquid-gated FET UA biosensors for 0.1 mM UA, (**b**) *I*_D_–*V*_G_ response of a liquid-gated FET UA biosensor for 0.1 mM UA up to 4 weeks of storage, (**c**) *I*_D_–*V*_G_ response of a liquid-gated FET UA biosensor for a serum sample only and serum sample containing known low (0.1 mM) and high (2.0 mM) concentrations of UA, and (**d**) histogram of estimated UA concentrations.

Fabrication reproducibility was evaluated by making four liquid-gated FET-based UA biosensors with the same protocol. All four biosensors exhibited consistent sensing behavior toward UA in PBS buffer, demonstrating excellent reproducibility with a low relative standard deviation (RSD) of 5.6% (Figure 5a). To further examine storage stability, the FET-based UA biosensors were tested immediately after fabrication and then stored at 4 °C for 4 weeks (Figure 5b). The sensing responses recorded over this period indicate that the FET-based UA biosensor retained over 93% of its original response after four weeks, confirming its good long-term stability and durability under refrigerated conditions. These findings demonstrate that the liquid-gated FET-based UA biosensor possesses excellent selectivity, fabrication reproducibility, and storage stability, underscoring its reliability for practical sensing applications. Moreover, the liquid-gated FET-based UA biosensor fabrication platform offers significant potential for further optimization, particularly through surface modification and functionalization strategies that could enhance enzyme immobilization efficiency, signal transduction, and overall device performance.

### 3.4. Practical Applicability

To evaluate the practical applicability of the fabricated liquid-gated FET-based UA biosensor, a human serum sample (Sigma-Aldrich) was analyzed using a standard spiking method. Known low (0.1 mM) and high (2.0 mM) concentrations of UA were spiked into human serum. The UA concentrations were measured with a fabricated FET biosensor (Figure 5c). The estimated UA concentrations are presented as a histogram in Figure 5d. The recovery (%) and RSD (%) were calculated (Table 2). The FET-based UA biosensor exhibited high recovery and low RSD, demonstrating its reliability and suitability for UA detection in real serum matrices.

## 4. Conclusions

In conclusion, liquid-gated FET-based UA biosensors were successfully fabricated by vertically growing ZnO NRs on the transistor’s seeded channel, followed by immobilization of uricase. Vertically grown ZnO NRs offer significantly greater surface area and structural stability, providing an improved platform for efficient attachment of the uricase enzyme. The resulting uricase-ZnO NR-based liquid-gated FET-based UA biosensor exhibited high sensitivity (12.45 μA·mM^−1^·cm^−2^) and a low LOD of ~0.0043 mM, along with a broad linear detection range (0.05–2.75 mM). Such an extended linear range is particularly advantageous for practical and on-site UA monitoring. Moreover, the FET biosensor demonstrated excellent selectivity toward UA, confirming its potential for real-sample analysis. Reproducibility tests revealed consistent fabrication performance, while storage stability studies showed that the device retained over 93% of its original response after four weeks, highlighting its durability. The reproducible, straightforward fabrication process offers a promising route to the scalable production of ZnO NR-based liquid-gated FET-based UA biosensors. Furthermore, this fabrication strategy can be readily adapted to develop highly sensitive liquid-gated FET-based biosensors tailored for the detection of specific analytes.

## Figures and Tables

**Table 1:** Comparative biosensing performance of the liquid-gated FET-based UA biosensors with previous reports.

Biosensor	Detection Method	Linear Range (mM)	Sensitivity (μA·mM^−1^·cm^−2^)	LOD (μM)	Ref.
GCE/Cu_2_O/Fc/uricase/Nf	DPV	0.0001–1	1.9	0.06	[49]
SPE-Au/DNA probes	SWV	0.00001–0.1	-	0.25	[50]
GCE/Au@Pd-rGO	DPV	0.0001–0.35	-	0.02	[51]
SPCE/uricase	CV	0–0.48	-	6	[52]
PET/PyTS@Ti_3_C_2_Tx	Amp	0.005–0.1	-	0.48	[53]
Si/Ag/ZnO NSs/uricase/Nf	Amp	0.05–2	129.81	5	[54]
PI/CuNi-MOF@rGO	Amp	0.01–1	26	9.09	[55]
SPE/AuNPs-TiS_2_/uricase/Nf	Amp	0.005–2	220.8	0.18	[56]
PET/PBG ink/uricase	Amp	0–1	2.32	-	[57]
Si/SiO_2_/ZnO NRs/uricase/Nf	FET	0.05–2.75	12.45	~4.3	This work

Abbreviations: GCE, glassy carbon electrode; Cu_2_O, cuprous oxide; Fc, ferrocene; DPV, differential pulse voltammetry; Au, gold; SPE, screen-printed electrode; SWV, square wave voltammetry; Si, silicon; Ag, silver; NSs, nanosheets; rGO, reduced graphene oxide; CV, cyclic voltammetry; PET, polyethylene terephthalate; Amp, amperometric; PI, polyimide; PBG, Prussian-blue graphite.

**Table 2:** UA detection in human serum using our fabricated liquid-gated FET-based UA biosensor.

Real Sample	Spiked (mM)	Detected (mM)	Recovery (%)	RSD (%) (*n* = 4)
Human serum (H4522)	0	0.27	-	-
	0.1	0.3678	97.8	4.8
	2.0	2.256	99.3	5.6

## Data Availability

The original contributions presented in this study are included in the article. Further inquiries can be directed to the corresponding authors.
